# Outcomes among oropharyngeal and oral cavity cancer patients treated with postoperative volumetric modulated arctherapy

**DOI:** 10.3389/fonc.2023.1272856

**Published:** 2023-10-31

**Authors:** Cécile Mione, Mélanie Casile, Juliette Moreau, Jessica Miroir, Ioana Molnar, Emmanuel Chautard, Maureen Bernadach, Myriam Kossai, Nicolas Saroul, F. Martin, Nathalie Pham-Dang, Michel Lapeyre, Julian Biau

**Affiliations:** ^1^ Department of Radiation Therapy, Centre Jean Perrin, Clermont-Ferrand, France; ^2^ INSERM U1240 IMoST, University of Clermont Auvergne, Clermont-Ferrand, France; ^3^ UMR 501, Clinical Investigation Centre, Clermont-Ferrand, France; ^4^ Department of Clinical Research, Clinical Search and Innovation, Centre Jean Perrin, Clermont-Ferrand, France; ^5^ Medical Oncology Department, Jean Perrin Center, Clermont-Ferrand, France; ^6^ Department of Pathology and Molecular Pathology, Centre Jean Perrin, Clermont-Ferrand, France; ^7^ Department of Otolaryngology-Head and Neck Surgery, Clermont-Ferrand University Hospital, Clermont-Ferrand, France; ^8^ Department of Maxillo-Facial Surgery, Clermont-Ferrand University Hospital, Clermont-Ferrand, France

**Keywords:** head and neck cancer, radiotherapy, post-operative, VMAT, recurrences

## Abstract

**Background:**

Presently, there are few published reports on postoperative radiation therapy for oropharyngeal and oral cavity cancers treated with IMRT/VMAT technique. This study aimed to assess the oncological outcomes of this population treated with postoperative VMAT in our institution, with a focus on loco-regional patterns of failure.

**Material and methods:**

Between 2011 and 2019, 167 patients were included (40% of oropharyngeal cancers, and 60% of oral cavity cancers). The median age was 60 years. There was 64.2% of stage IV cancers. All patients had both T and N surgery. 34% had a R1 margin, 42% had perineural invasion. 72% had a positive neck dissection and 42% extranodal extension (ENE). All patients were treated with VMAT with simultaneous integrated boost with three dose levels: 66Gy in case of R1 margin and/or ENE, 59.4-60Gy on the tumor bed, and 54Gy on the prophylactic areas. Concomittant cisplatin was administrated concomitantly when feasible in case of R1 and/or ENE.

**Results:**

The 1- and 2-year loco-regional control rates were 88.6% and 85.6% respectively. Higher tumor stage (T3/T4), the presence of PNI, and time from surgery >45 days were significant predictive factors of worse loco-regional control in multivariate analysis (p=0.02, p=0.04, and p=0.02). There were 17 local recurrences: 11 (64%) were considered as infield, 4 (24%) as marginal, and 2 (12%) as outfield. There were 9 regional recurrences only, 8 (89%) were considered as infield, and 1 (11%) as outfield. The 1- and 2-year disease-free survival (DFS) rates were 78.9% and 71.8% respectively. The 1- and 2-year overall survival (OS) rates were 88.6% and 80% respectively. Higher tumor stage (T3/T4) and the presence of ENE were the two prognostic factors significantly associated with worse DFS and OS in multivariate analysis.

**Conclusion:**

Our outcomes for postoperative VMAT for oral cavity and oropharyngeal cancers are encouraging, with high rates of loco-regional control. However, the management of ENE still seems challenging.

## Introduction

Surgery is one of the cornerstone treatments for oropharyngeal and oral cavity cancers (1). Adjuvant postoperative radiation therapy is recommended for patients with adverse features, including advanced disease and inadequate margins. The addition of concomitant chemotherapy is recommended, particularly for patients who have ‘high risk’ pathological features including extranodal extension (ENE) and/or a positive surgical margin (1, 2).

Intensity Modulated Radiation Therapy (IMRT) or Volumetric Modulated Arctherapy (VMAT) is today the recommended radiation technique for the treatment of head and neck cancers (3). IMRT/VMAT for head and neck cancers is a complex technique both for target volume delineation and treatment planning (4, 5). The delineation of the target volumes is an essential step conditioning the results of the treatment, particularly in terms of loco-regional control (6–9). Presently, there are few published reports on postoperative radiation therapy for oropharyngeal and oral cavity cancers treated with IMRT/VMAT technique. This study aimed to assess the oncological outcomes of operated patients with oropharyngeal and oral cavity squamous cell carcinomas (SCC), treated with postoperative VMAT in our institution, with a focus on loco-regional patterns of failure.

## Materials and methods

### Patients

The database maintained by the Department of Radiation Oncology at our institution was used to identify patients treated with postoperative VMAT for oropharyngeal or oral cavity SCC from May 2011 to December 2019. Patients with distant metastases or concomitant malignancies at the time of diagnosis, histology other than SCC, R2 margins, and/or a previously irradiated cancer of the head and neck were excluded.

One hundred and sixty-seven patients were retrospectively reviewed. Patient characteristics are described in **Table 1**.

**Table 1:** Patient and disease characteristics.

Characteristics	N (%)
Gender	
Male	122 (73)
Female	45 (27)
**Median Age (range)**	60 (20-94)
Site	
Oral Cavity	100 (60)
Oropharynx	67 (40)
Tonsil	48 (29)
Base of tongue	15 (9)
Soft palate	4 (2)
T-stage	
1-2	72 (43)
3-4	95 (57)
**N-stage**	
0-1	72 (43)
2-3	95 (57)
UICC Stage 2009	
II	23 (13,7)
III	37 (22,1)
IVa	94 (56,4)
IVb	13 (7,8)
p16	
Positive	24 (14)
Negative	38 (23)
Unspecified	105 (63)
**Smokers**	125 (75)
WHO status	
0-1	151 (90)
2-3	16 (10)
Treatment	N (%)
Chemotherapy	
Concomitant	72 (43)
Neoadjuvant	5 (3)
No	90 (54)
Nodal surgery	
Bilateral	90 (53.9)
Unilateral	77 (46.1)
Tumor characteristics	N (%)
**Extranodal extension+**	68 (42)
Tumor differentiation	
Well	74 (44)
Moderate	56 (34)
Poor	19 (11)
Unspecified	18 (11)
Lymphovascular invasion	
Yes	48 (29)
No	117 (70)
Unspecified	2 (1)
Perineural invasion	
Yes	70 (42)
No	91 (54)
Unspecified	6 (4)
Margin	
R1	56 (34)
R0 close < 5mm	63 (37)
R0	41 (25)
Unspecified	7 (4)

The initial location of the tumors was the oropharynx in 40% of cases (n=67) and the oral cavity in 60% (n=100) of cases. The median age of the patients was 60 years (20 - 94 years). One hundred and twenty-five patients were smokers (75%). The WHO performance status was assessed as 0 for 78 patients (46,7%), 1 for 73 patients (43,7%), 2 for 15 patients (8,9%), and 3 for 1 patient (0,7%). The TNM stages (UICC 2009) were as follows: there were 23 stage II (13.7%), 37 stage III (22.1%), 94 stage IVa (56.4%), and 13 stage IVb (7.8%).

### Treatment

The overall treatment strategies were individualized for each patient following recommendation by a multidisciplinary tumor board.

#### Surgery

All patients included underwent surgery on the primary tumor accompanied by lymph node dissection (unilateral for 46.1% and bilateral for 53.9%). The operative technique depended on the location and initial extension of the disease. Sixty-seven patients (40%) had a free flap inserted during surgical reconstruction.

#### Anatomic pathology

Fifty-six patients (34%) had a positive R1 margin, and sixty-three (37%) had a close margin (<5mm). Seventy patients (42%) had peri-neural invasion (PNI), and 48 (29%) had lympho-vascular invasion. One hundred and twenty one patients (72%) had positive neck dissections, and 68 patients (42%) had ENE (**Table 1**).

#### Radiotherapy

Patients underwent radiotherapy in case of advanced tumors (T3-T4), close or positive margins, lymph node involvement with or without ENE, the presence of lymphatic-vascular space invasion, and/or PNI (10, 11). The median interval from surgery to initiation of radiotherapy was 46 days (24 - 100). All patients were irradiated with VMAT (Rapidarc®, Varian Medical Systems, Palo Alto, CA, USA) with simultaneous integrated boost (SIB). A planning CT-scan, supine, in the treatment position, was performed, with 2.5-mm-thick slices, and a personalized thermoformed mask with 5 attachment points. Preoperative imaging was merged with the planning CT scan to guide the contouring of this surgically reshaped area. Target volumes and organs at risk were delineated according to the different expert recommendations (12–17). Three clinical target volumes (CTV) were typically defined. CTV1 (59.4 to 60 Gy) was defined as the preoperative tumor bed with a margin (1 to 2 cm), and manually adjusted according to anatomical barriers. CTV2 (54 Gy) was defined as subclinical tumor sites at risk, according to the risk of tumor involvement (14–16), and as prophylactic nodal irradiation, following guidelines (12, 13, 15). CTV3 (66 Gy) was occasionally identified, in cases of ENE and/or positive margin. Planning target volumes (PTV) related to positioning errors and movements were obtained by adding a 4 mm margin around the CTVs. Treatment was delivered in 30-33 fractions.

The treatment planning system was Eclipse® (Varian Medical Systems). Treatment plans followed the recommendations of the International Commission of Radiation Units report n°83 (18). The treatment was delivered using a Clinac IX® or Novalis TX® (Varian Medical Systems) linear accelerators delivering 6-MV photons, with daily position control by KV/KV beams or CBCT.

#### Chemotherapy

Seventy-two patients (43%) underwent concomitant chemotherapy. The main indications were R1 positive margins and/or ENE among patients under 70 years of age and in the absence of contraindications (10, 11, 19). Concomitant chemotherapy protocols included either three-weekly high dose Cisplatin, or weekly Cisplatin (**Table 1**). Five patients (3%) received neoadjuvant chemotherapy with Taxotere-Cisplatin-5Fluoro-Uracile.

### Follow-up

Weekly evaluations were performed by the radiation oncologist for all patients during radiation treatment. The first post-treatment follow-up was at 3 months after radiation completion. Then, every 3 months for the first and the second year, alternating surgeon and radiation oncologist, and at least twice a year for up to 5 years, and thereafter yearly. A follow-up imaging was performed at 3 months and then annually.

### Recurrences

Local and regional recurrences were confirmed via radiologic imaging (i.e. progression in subsequent images or high SUV on PET imaging) or via pathology specimens (i.e. from surgical biopsy). Diagnostic contrast-enhanced CT and/or PET/CT or MRI documenting the initial evidence of local recurrence were investigated. Radiologically evident recurrence volumes were manually segmented and reviewed by four experienced radiation oncologists (CM, JMO, JMI and JB). The corresponding original planning CTs were identified and the original plans were restored. Recurrence CT was co-registered with initial planning CT using a deformable image registration (Aria with MIRS application, version 2.1, Varian Medical Systems) (20, 21). The recurrence volume was transferred to the initial planning CT and was subsequently deformed according to the deformable co-registration. A clinical validation was carried out by the radiation oncologists. The most likely point of origin of the recurrence was defined clinically by the radiation oncologists, based on their knowledge of anatomy and cancer spread pathways according to Due et al. (22, 23). If the point of origin of the recurrence was outside the initial target volume, recurrence was considered to be “outfield”; if the point of origin of the recurrence was inside the initial target volume, recurrence was considered to be “infield”; and if the point of origin of the recurrence was on the boundary of the initial target volume, recurrence was considered to be “marginal”.

### Statistical analysis

Groups defined by initial tumor localization were compared using Fisher’s exact test and the Wilcoxon-Mann-Whitney test. The Kaplan-Meier method was used to calculate survival curves. The last day of radiation therapy was used as time zero. Comparisons between survival curves were made using the log-rank test. Median follow-up was estimated using the reverse Kaplan-Meier method. Factors associated with survival were analyzed using univariate Cox regression models followed by penalized multivariate models, obtained by including all variables with a p-value <.05 in the univariate analysis and model selection with the LASSO method. All analyses were performed using R statistical software version 4.1.0 (R-Project, GNU GPL). P-values under 0.05 were considered significant.

The factors associated with survival analyzed were: gender (male/female), tobacco use (never/current or stopped), WHO stage (0/1-3), tumor status (T1-T2/T3-T4), number of pathological lymph nodes (≥3/<3), tumor differentiation (good/moderate, poor), lymphovascular invasion, PNI, ENE, margin status (R0/close, R1), radiotherapy-treatment time and surgery-radiotherapy time.

## Results

### Local, regional and loco-regional control

The median follow-up was 33 months. During follow-up, 26 patients (16%) developed loco-regional recurrences: 12 local, 9 regional, and 5 both local and regional (**Table 2**).

**Table 2:** Prognostic and predictive factors.

	Prognostics factors	Univariate Analysis HR (CI 95%)	Multivariate Analysis HR (CI 95%)
**Local** **control**	T3-T4	14.135 (1.873 – 106.702) p<0.001	9.88 (1.296 – 75.315) p=0.03
	PNI +	6.633 (1.887 – 23.323) p<0.001	5.127 (1.453 – 18.089) p=0.01
	Oral cavity	1.277 (1.079 - 1.965) p=0.02	1.47 (1.132 – 1.669) p=0.24
**Regional control**	Lymph node >3	4.906 (1.581 – 15.225) p=0.009	4.906 (1.581 – 15.225) p=0.006
**Loco-regional control**	T3-T4	3.979 (1.504 – 10.524) p=0.002	3.172 (1.167 – 8.624) p=0.02
	Time from surgery to RT	1.035 (1.008 – 1.062) p=0.03	1.041 (1.003 – 1.082) p=0.04
	PNI +	2.911 (1.294 – 6.549) p=0.008	2.714 (1.17 – 6.296) p=0.02
	Oral cavity	1.362 (1.146 - 1.898) p=0.02	1.581 (1.229 – 1.477) p=0.25
**Metastasis-free** **survival**	PNI+	2.213 (0.992 -4.936) p=0.049	1.939 (0.853 – 4.407) p=0.11
	ENE +	3.549 (1.543-8.165) p=0.002	3.498 (1.506 – 8.122) P=0.0044
**Disease-free survival**	T3-T4	2.551 (1.409 – 4.617) p=0.001	2.544 (1.399 – 4.625) p=0.002
	ENE +	1.785 (1.055 – 3.02) p=0.03	1.921 (1.127 – 3.273) p=0.02
**Overall** **survival**	WHO stage 1-3	1.997 (1.067 – 3.74) p=0.03	1.651 (0.868 – 3.14) p=0.13
	T3-T4	2.606 (1.308 – 5.192) p=0.004	2.529 (1.252 – 5.111) p=0.01
	ENE +	2.167 (1.17 – 4.013) p=0.01	2.295 (1.213 – 4.345) p=0.01

PNI +, presence of perineural invasion, RT, radiation therapy, ENE +, presence of extranodal extension.

The 1 and 2-year local control rates were 93% and 90% respectively for the overall population. For oral cavity cancers, the 2-year local control rate was 85.1% vs 96.7% for oropharyngeal cancers (p=0.031). Higher tumor stages (T1-2 vs T3-4; p< 0.001) and presence of PNI (p<0.001) were predictive factors for poorer local control in univariate and multivariate analysis (p=0.03 and p=0.01 respectively) (**Table 3**).

**Table 3:** Initial characteristics and analysis of the patients who developed a local and/or regional recurrence.

	Patients	TNM	Age at diagnosis	Location	Nodal Extension	Margin	PNI	Flap	Chemotherapy	Surgery-RT interval (days)	Prescribed Dose (Gy)	Treatment Time (days)	Time to recurrence (months)	Recurrence anatomical description	Recurrence analysis
**Local T recurrence only**	**1**	T4aN2aM0	52	Mobile tongue	ENE +	R1	Yes	Yes	Yes	39	66	46	9.2	Tongue/tongue flap junction	Infield.
	**2**	T3N2cM0	78	Base of tongue	ENE +	R1	Yes	Yes	No	41	66	50	7.1	Base of Tongue	Infield.
	**3**	T4aN0M0	50	Mandible	N0	R1	Yes	No	No	53	66	50	20.7	Homolateral mandible	Infield.
	**4**	T4aN2cM0	54	Floor of mouth	ENE +	R1	Yes	Yes	Yes	49	66	49	15.7	Fibula flap region	Infield
	**5**	T3N1M0	77	Mobile tongue	ENE -	R0	No	No	No	47	66	45	16.4	Mobile tongue	Infield.
	**6**	T3N1M0	59	Floor of mouth	ENE -	R1	Yes	No	Yes	48	66	46	4.2	Mobile tongue/ floor of mouth junction	Infield
	**7**	T4N1M0	72	Retromolar area	ENE +	R<5 mm	Yes	Yes	No	51	66	48	12.3	Digastric muscle	Outfield
	**8**	T4aN0M0	62	Floor of mouth	N0	R<5 mm	Yes	Yes	No	48	60	38	2.7	Fibula flap region	Marginal
	**9**	T3N1M0	60	Upper lip	ENE +	R0	Yes	Yes	Yes	83	60	48	3.1	Upper jaw	Infield.
	**10**	T3N0M0	56	Base of tongue	N0	R1	Yes	Yes	Yes	38	66	47	29.1	Macroscopic PNI along the lingual nerve	Infield
	**11**	T1N0M0	65	Tonsil	N0	R<5 mm	Yes	No	No	39	66	45	4.8	Mobile Tongue	Marginal
	**12**	T3N0M0	79	Hard Palate	N0	R1	Yes	No	No	41	66	44	6.6	Hard Palate	Marginal
**Both local and regional recurrence**	**13**	T4aN2cM0	42	Floor of mouth	ENE +	R<5 mm	Yes	Yes	Yes	48	60	38	4.7	T: Tongue / flap junction	T: Infield.
	**14**	T3N0M0	77	Cheek	N0	R1	No	Yes	No	35	66	45	27.8	T: Mandible	T: Infield
	**15**	T4aN2bM0	65	Retromolar area	ENE -	R1	No	Yes	Yes	41	66	43	11	T: Infratemporal fossa	T: Outfield
	**16**	T4aN2cM0	26	Mobile tongue	ENE +	R1	Yes	Yes	Yes	35	66	45	1.4	T: Base of tongue	T: Infield.
	**17**	T4N2cM0	20	Floor of mouth	ENE +	R<5 mm	Yes	Yes	Yes	41	60	45	3	T: Oropharynx Hypopharynx	T: Marginal
**Regional N recurrence only**	**18**	T4aN2cM0	53	Mobile Tongue	ENE + III controlateral	R1	Yes	Yes	Yes	42	66	48	4	IVa controlateral	Infield (54Gy)
	**19**	T3N0M0	51	Retromolar area	N0	R0		Yes	No	49	60	50	3.8	Ib homolateral	Infield (54Gy)
	**20**	T2N2cM0	54	Oropharynx	ENE + IIB homolateral	R1		No	Yes	42	66	51	39	VIIA homolateral	Infield (54Gy)
	**21**	T3N2bM0	80	Mobile tongue	ENE + IIA homolateral	R<5mm		No	No	44	66	47	4	IV homolateral	Infield (54Gy)
	**22**	T1N2aM0	74	Left Tonsil	ENE + IIB homolateral	R0	No	No	No	43	66	44	12	VIIA homolateral	Infield (60Gy)
	**23**	T4aN3bM0	79	Hard Palate	ENE + IIA homolateral	R1	Yes	No	No	48	60	39	8.5	II A IIB III contralateral (unilateral irradiation)	Outfield
	**24**	T1N2cM0	49	Mobile tongue	ENE + IIA bilateral	R1		No	Yes	44	66	48	4	IB homolateral	Infield (60Gy)
	**25**	T2N2bM0	56	Floor of mouth	ENE + IIA homolateral	R<5 mm	Yes	Yes	Yes	43	66	50	9	IIB-IV-V homolateral	Infield (60Gy)
	**26**	T4N1M0	80	Floor of mouth	ENE – IIB homolateral	R1	Yes	Yes	No	43	60	41	4	IIB homolateral	Infield (60Gy)

R1= positive margin, R<5mm=close margin <5mm, R0=negative margin, ENE +, extranodal extension; ENE -, positive node without extranodal extension; N - , no positive node; Surgery – RT interval, interval between surgery and the first day of radiotherapy.

The 1 and 2-year regional control rates were 93.1%. A number of lymph node over 3 was the only predictive factor for a poorer regional control in univariate and multivariate analysis (p=0.009 and p=0.006 respectively) (**Table 3**).

**Figure 1:**
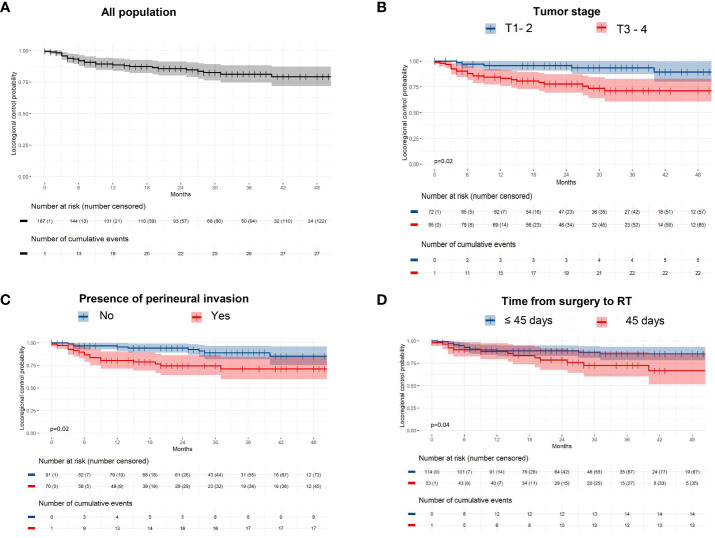
Loco-regional control among **(A)** the entire population, and **(B)** according to tumor staging, **(C)** the presence of perineural invasion, and **(D)** Time from surgery to radiotherapy.

The 1- and 2-year loco-regional control rates were 88.6% and 85.6% respectively (**Figure 1**), with 85.3% and 80.1% among oral cavity cancer and 93.6% and 93.6% among oropharynx cancer (p=0.022). Higher tumor stages (T1-2 vs T3-4; p=0.002), presence of PNI (p=0.008) and time from surgery to initiation of VMAT (p=0.03) were predictive factors for poorer loco-regional control in univariate and multivariate analysis respectively (p=0.02, p=0.04, and p=0.02 respectively; **Table 3**; **Figure 1**).

### Local and regional patterns of recurrence

Seventeen patients (10%) developed a local recurrence and 13 patients (8%) a regional recurrence, of whom 5 patients had both a local and regional recurrence. The initial characteristics and patterns of recurrence are described in **Table 2**.

Concerning the 17 local recurrences, 11 (64%) were considered as infield, 4 (24%) as marginal, and 2 (12%) as outfield. Concerning the 9 regional recurrences only, 8 (89%) were considered as infield, and 1 (11%) as outfield.

### Metastasis

**Figure 2:**
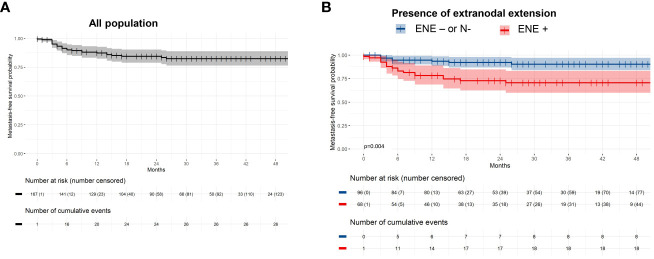
Metastasis-free survival among **(A)** the entire population, and **(B)** according to the presence of extranodal extension.

During follow-up, there were 26 metastatic events (15.6%) with a 2-year metastasis-free survival probability of 84.5% (**Figure 2**). Ten patients treated for an oropharyngeal cancer (2-year probability of metastasis-free survival = 85.9%) and 16 patients treated for an oral cavity tumor (2-year probability of metastasis-free survival = 83.3%) developed secondary localizations (bone, lung, cutaneous and liver) with no difference according to the initial tumor location (p=0.74). In univariate analysis, PNI and ENE were associated with poorer metastasis free survival (p=0.049, p=0.002). In multivariate analysis, only ENE remained significant (p=0.004; **Table 3**; **Figure 2**).

### Survival

**Figure 3:**
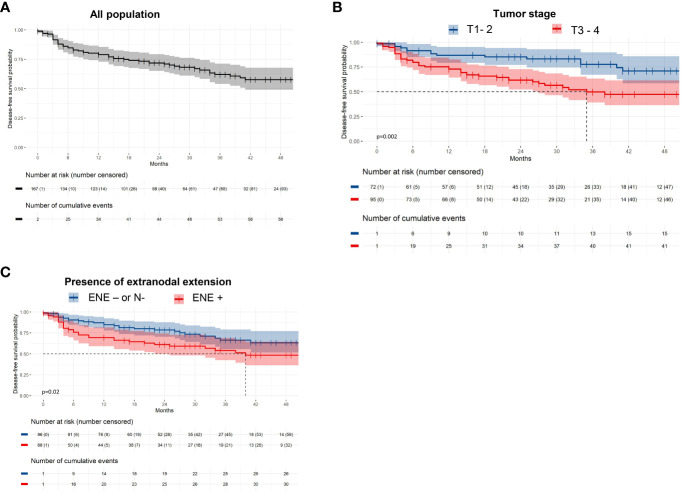
Disease-free survival among **(A)** the entire population, and **(B)** according to tumor staging, and **(C)** the presence of extranodal extension.

The 1- and 2-year disease-free survival (DFS) rates were 78.9% and 71.8% respectively (**Figure 3**). Higher tumor stages (T1-2 vs T3-4; p= 0.001) and presence of ENE (p=0.03) were unfavorable prognostic factors for DFS in univariate and multivariate analysis (p=0.002 and p=0.02 respectively; **Table 3**; **Figure 3**).

**Figure 4:**
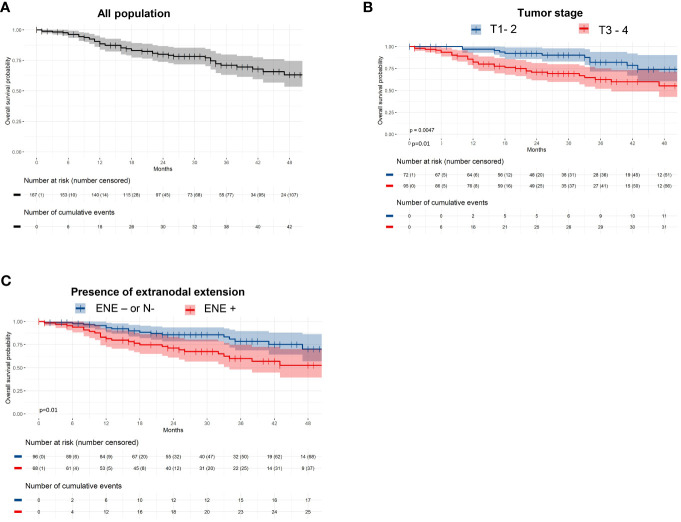
Overall survival among **(A)** the entire population, and **(B)** according to tumor staging, and **(C)** the presence of extranodal extension.

Forty-two patients (25%) died during follow-up. The 1- and 2-year overall survival (OS) rates were 88.6% and 80% respectively (**Figure 4**). In univariate analysis, WHO stage ≥1 (p = 0.03), presence of ENE (p = 0.01) and T3-T4 tumors (p=0.004) were unfavorable prognostic factors for OS. In multivariate analysis, the presence of ENE and T3-T4 tumors were independent unfavorable prognostic factors for OS (p=0.01 and p=0.01 respectively; **Table 3**; **Figure 4**).

## Discussion

IMRT/VMAT has been increasingly used over the last two decades for the treatment of head and neck cancers, and is today the gold standard technique in radiation therapy for these cancers. However, the data regarding the outcomes associated with IMRT/VMAT in the post-operative setting for oral cavity and oropharyngeal cancers is very limited (24–31). Thus our series, despite its relatively small number of patients (n=167), is one of the largest reported to date. The outcomes reported in our series are in line with those reported in the literature (25, 26, 29, 31–34) (**Table 4**). We found that postoperative VMAT reached high rates of local and loco-regional control. We found that the presence of ENE, despite present-day radiochemotherapy techniques, remains a major issue.

**Table 4:** Outcomes in previous reports concerning post-operative IMRT in oral cavity and oropharynx SCC.

Authors	Study Period	Sites (No. of patients)	Median Follow-up (months)	LR recurrence (No., %)	Local recurrence (No., %)	2-year LC rate	2-year LRC rate	2-year OS rate	Risk factors (in multivariate analysis)
**Quilan-Davidson et al.** (24)	2000-2012	289 OC	35	63 (22%)	31 (11%)	86%	79%	69%	–**LC recurrence:** DOI>1.5mm, R1, tobacco <320PY, no free flap reconstruction, –**LR recurrence:** DOI, R1, no free flap reconstruction, neck dissection status, and tobacco < 20PY –**OS:** neck dissection status, lymphovascular invasion, DOI
**Chan et al.** (26)	2005-2010	180 OC	34	38 (21%)	22 (12%)	87%	78%	65%	–**LC recurrence:** Oral tongue subsite, –**LR recurrence:** N2 –**OS:** Oral tongue subsite, N2, R1, tumor size
**Collan et al.** (29)	2001-2007	40 OC 62 OP	55	8 (8%): 5 OC et 3 OP	3 (3%)	OC: 85% OP :94%	NR	OC:82% OP: 96%	–**LC recurrence:** Oral cavity site, –**OS:** Oral cavity site
**Present study**	2011-2019	100 OC 67 OP	33	26 (15%)	17 (10%)	OC: 85.1% OP: 96.7%	85.6%	OC: 77.3% OP: 83.8%	–**LC recurrence:** T3-T4 stage, ENE + –**LR recurrence:** T3-T4, time from surgery to RT, PNI –**OS:** T3-T4 stage, ENE +

OC oral cavity, OP, oropharynx, LC, local control, LRC, loco-regional control, OS, overall survival, NR, not reported, DOI, depth of invasion, PY, pack-year, R1, positive margins.

There are a few limitations to our study that need to be highlighted. Potential biases, inherent in any retrospective analysis, could have affected the results of this study. Because of the retrospective nature of the study, certain data missing in the medical files could not be assessed for all patients. For example, the depth of invasion for oral cavity cancers was often missing, not allowing to use the latest UICC classification. The same issue was faced regarding HPV status, which was not available for all patients, particularly those treated the earliest in the cohort. We also found that the collection of all toxicities were not robust enough to allow good quality interpretation of the data, so we only focused on the oncological outcomes. This series was also single-center. However, all patients were treated in the same institution with surgery and post-operative radiotherapy in fairly homogeneous manner for both dose prescription and delineation.

Concerning local recurrences, during follow-up only 17 patients (10%) developed a local recurrence with actuarial 1 and 2-year local control rates of 93% and 90% respectively. The vast majority of these local recurrences were found to be *infield*. Higher tumor stages and presence of PNI were the only predictive factors in multivariate analysis for poorer local control. R1 margins were not found to be predictive factors for local control. This is in line with various recent reports that have also found that R1 margins were no longer a factor of poorer local control since the emergence of radiochemotherapy (24, 27, 29, 35). It seems that the negative impact of R1 margins has been fully negated by using higher radiotherapy doses (usually 66Gy) and radiochemotherapy. However, this does not seem to be the case for ENE. Indeed, in our series, the presence of ENE was found to be a prognostic factor for poorer DFS, metastasis-free survival, and OS. A recent series of 439 patients with head and neck SCC (all localizations) treated with postoperative radiotherapy also reported the negative prognostic value of ENE despite the use of radiochemotherapy (35). This finding highlights the need to find new strategies for patients with ENE treated with postoperative radiochemotherapy, as in the ongoing NIVOPOSTOP trial testing the addition of Nivolumab to standard cisplatin-based chemo-radiation (NCT03576417).

We also found that oral cavity cancers generally had poorer outcomes than oropharyngeal cancers, with 2-year LC rates of 85.1% vs 96.7% (p=0.031), and 2-year OS rates of 77.3% vs 83.8% (non-significant, probably due to a lack of statistical power). This is in line with previously reported series (25, 29, 36).

Regarding chemotherapy, 72 patients (43%) were treated with radiotherapy combined with chemotherapy. The local control and OS rates for these high-risk patients were similar to those for patients with a lower risk of recurrence, treated without concomitant chemotherapy. It is possible that without concomitant chemotherapy the outcome among high-risk patients would have been worse (29). The total number of failures in this series is however too small to enable any robust calculations between subgroups.

## Conclusion

Our outcomes for postoperative VMAT for oral cavity and oropharyngeal cancers are very encouraging, with high rates of loco-regional control (85.6% at 2 years). However, the management of ENE still seems challenging, as these events were identified as highly unfavorable prognostic factors.

## Data availability statement

The raw data supporting the conclusions of this article will be made available by the authors, without undue reservation.

## Ethics statement

This study was approved by CECIC Rhône-Alpes-Auvergne. All patients were informed and were free to oppose their participation in this study. The studies were conducted in accordance with the local legislation and institutional requirements. The ethics committee/institutional review board waived the requirement of written informed consent for participation from the participants or the participants’ legal guardians/next of kin because All patients were informed and were free to oppose their participation in this study.

## Author contributions

JB: Conceptualization, Methodology, Supervision, Writing – original draft, Writing – review & editing. CM: Conceptualization, Investigation, Methodology, Writing – original draft. MC: Conceptualization, Methodology, Writing – review & editing. JMo: Data curation, Methodology, Writing – review & editing. JMi: Data curation, Methodology, Writing – review & editing. IM: Conceptualization, Formal Analysis, Methodology, Writing – review & editing. EC: Conceptualization, Formal Analysis, Methodology, Writing – review & editing. MB: Investigation, Methodology, Writing – review & editing. MK: Investigation, Methodology, Writing – review & editing. NS: Investigation, Methodology, Writing – review & editing. NP-D: Investigation, Methodology, Writing – review & editing. FM: Investigation, Methodology, Writing – review & editing. ML: Conceptualization, Methodology, Supervision, Validation, Writing – review & editing.
